# Detrimental effect of anemia after mechanical thrombectomy on functional outcome in patients with ischemic stroke

**DOI:** 10.3389/fneur.2023.1299891

**Published:** 2023-12-19

**Authors:** Ryoma Inui, Junpei Koge, Kanta Tanaka, Takeshi Yoshimoto, Masayuki Shiozawa, Soichiro Abe, Hiroyuki Ishiyama, Hirotoshi Imamura, Jin Nakahara, Hiroharu Kataoka, Masafumi Ihara, Kazunori Toyoda, Masatoshi Koga

**Affiliations:** ^1^Department of Cerebrovascular Medicine, National Cerebral and Cardiovascular Center, Osaka, Japan; ^2^Department of Neurology, Keio University School of Medicine, Tokyo, Japan; ^3^Division of Stroke Care Unit, National Cerebral and Cardiovascular Center, Osaka, Japan; ^4^Department of Neurology, National Cerebral and Cardiovascular Center, Osaka, Japan; ^5^Department of Neurosurgery, National Cerebral and Cardiovascular Center, Osaka, Japan

**Keywords:** ischemic stroke, large vessel occlusion, endovascular therapy, mechanical thrombectomy, anemia

## Abstract

**Background:**

Anemia can occur due to an aspiration maneuver of blood with thrombi during mechanical thrombectomy (MT) for stroke. However, the association between postoperative anemia and stroke outcomes is unknown.

**Methods:**

In a registry-based hospital cohort, consecutive patients with acute ischemic stroke who underwent MT were retrospectively recruited. Patients were divided into the following three groups according to their hemoglobin (Hb) concentrations within 24 h after MT; no anemia (Hb concentrations ≥13 g/dL for men and ≥ 12 g/dL for women), mild anemia (Hb concentrations of 11–13 g/dL and 10–12 g/dL, respectively), and moderate-to-severe anemia (Hb concentrations <11 g/dL and < 10 g/dL, respectively). A 3-month modified Rankin Scale score of 0–2 indicated a favorable outcome.

**Results:**

Of 470 patients, 166 were classified into the no anemia group, 168 into the mild anemia group, and 136 into the moderate-to-severe anemia group. Patients in the moderate-to-severe anemia group were older and more commonly had congestive heart failure than those in the other groups. Patients in the moderate-to-severe anemia group also had more device passes than those in the other groups (*p* < 0.001). However, no difference was observed in the rate of final extended thrombolysis in cerebral infarction ≥2b reperfusion or intracranial hemorrhage among the groups. A favorable outcome was less frequently achieved in the moderate-to-severe anemia group than in the no anemia group (adjusted odds ratio, 0.46; 95% confidence interval, 0.26–0.81) independent of the baseline Hb concentration. A restricted cubic spline model with three knots showed that the adjusted odds ratio for a favorable outcome was lower in patients with lower Hb concentrations within 24 h after MT.

**Conclusion:**

Moderate-to-severe anemia within 24 h after MT is independently associated with a reduced likelihood of a favorable outcome.

**Clinical trial registration:**

https://www.clinicaltrials.gov, NCT02251665.

## Introduction

Advances in mechanical thrombectomy (MT) have dramatically improved the outcome of patients with acute large vessel occlusion (LVO). However, approximately half of the patients who undergo MT are functionally disabled or dead at 3 months (1). Research on perioperative management is essential to further improve the outcomes of MT (2, 3).

Anemia is found in approximately one quarter of patients with acute ischemic stroke on admission (4, 5). Previous studies have shown that moderate-to-severe anemia (hemoglobin [Hb] concentrations <11 g/dL) on admission are associated with poor functional outcomes after acute LVO (6). A recent cohort study showed that 75% of patients who underwent MT had anemia (Hb concentrations <13 g/dL for men and < 12 g/dL for women) 24 h after admission (7). Furthermore, a decrease in Hb concentrations during hospitalization was reported to be associated with a decreased likelihood of favorable outcomes (7). Post-MT anemia is likely to be a good target for intervention, but its prevention is more important.

An aspiration maneuver of blood with thrombi is essential during the MT procedure, regardless of the thrombectomy strategy (e.g., stent retriever alone (8), contact aspiration technique (9), or the combined technique) (10, 11), which may result in decreased postoperative Hb concentrations. Although a higher number of device passes may result in lower postoperative Hb concentrations, the relationship between the details of the MT procedure and postoperative anemia remains unclear.

We hypothesize that anemia caused by the blood aspiration maneuver during each device pass worsens functional outcomes. We aimed to investigate the association between Hb concentrations after MT and functional outcomes, while taking into consideration the details of the MT procedure.

## Methods

The data that support the findings of this study are available from the corresponding author on reasonable request.

### Study subjects

All patients with acute ischemic stroke admitted to our institute within 7 days from the last known well were prospectively registered in the National Cerebral and Cardiovascular Center Stroke Registry (12, 13). Consecutive patients who underwent MT as an endovascular revascularization procedure were enrolled in the present study from January 2014 to April 2022. Ethics approval was obtained from the local institutional review board (M23-073-7). Written informed consent was waived because clinical information obtained in routine clinical practice was used, no additional invasive procedures or costs were imposed on the patients, and the information was sufficiently anonymized. The National Cerebral and Cardiovascular Center Stroke Registry is registered with ClinicalTrials.gov (NCT02251665). The present study conforms to the Strengthening the Reporting of Observational Studies in Epidemiology guidelines for cohort studies (14).

### MT procedure

All endovascular procedures were performed by neurointerventionalists certified by the Japanese Society for Neuroendovascular Therapy in accordance with the American Heart Association/American Stroke Association Guidelines (15). Any devices for MT procedures available in Japan could be selected at the discretion of the treating physician. A balloon guide catheter was routinely used and navigated to the extracranial internal carotid artery as much as possible. MT procedures included stent retriever (SR) thrombectomy, contact aspiration (CA), or combined SR and CA (clot retrieval using SR and aspiration catheter as a unit) (16). All patients underwent MT under local anesthesia, and conscious sedation was added when required. Written informed consent for MT was obtained from each patient or a relative if the patient had communication difficulties. The reperfusion status after MT was assessed according to the extended thrombolysis in cerebral infarction (eTICI) scale (17). Intravenous thrombolysis was performed when indicated.

### Definition of anemia

Blood tests were usually performed at admission and within 24 h after MT. According to the cut-off points provided by the recent cohort study (18), patients were divided into the three following groups: no anemia, Hb concentrations ≥13 g/dL for men and ≥ 12 g/dL for women; mild anemia, Hb concentrations of 11–13 g/dL for men and 10–12 g/dL for women; and moderate-to-severe anemia, Hb concentrations <11 g/dL for men and < 10 g/dL for women.

### Clinical data collection

The following clinical information was collected: age, sex, body weight, premorbid modified Rankin Scale (mRS) score, baseline National Institutes of Health Stroke Scale (NIHSS) score, atrial fibrillation, cardiovascular risk factors (hypertension, diabetes mellitus, dyslipidemia, and current smoking), ischemic heart disease (history of myocardial infarction, angina, or coronary revascularization treatment), congestive heart failure, chronic kidney disease, active cancer (recurrent malignant tumor, metastases, or ongoing chemo−/radiotherapy) (19), previous stroke or transient ischemic attack, systolic blood pressure on admission, and laboratory data (Hb and creatinine concentrations). The ∆Hb represents the change in the Hb concentrations within 24 h after MT from the Hb concentration on admission. The estimated glomerular filtration rate (eGFR) was calculated according to the equation defined by the Japanese Society of Nephrology (20), and chronic kidney disease was defined as an eGFR below the threshold of 60 mL/min/1.73 m^2^ (21). The extent of ischemic change in the middle cerebral artery territory was graded using the Alberta Stroke Program Early Computed Tomographic Score (ASPECTS) on diffusion-weighted magnetic resonance imaging or non-contrast computed tomography. Intravenous thrombolysis was performed with alteplase at 0.6 mg/kg (dose approved in Japan) (22). Red blood cell (RBC) transfusions were performed at the discretion of the clinician.

### Outcomes

The procedural outcomes were the final eTICI scale, the first pass effect (23), the time from onset to groin puncture, the time from groin puncture to first successful recanalization with eTICI 2b–3, the time from groin puncture to final angiography, the total number of device passes, symptomatic intracranial hemorrhage (ICH) according to the definition of ECASS-II (24), any ICH within 36 h after onset, and the presence of subarachnoid hemorrhage. At 3 months, a favorable outcome (mRS score of 0–2), excellent outcome (mRS score of 0–1), favorable shift in the mRS score, and mortality were evaluated. In addition, neurological improvement, defined as a decrease of 10 or more points in the NIHSS score compared to the baseline or achieving a score of 0 at 7 days after the onset, and neurological deterioration, which was defined as an increase of 4 or more points in the NIHSS score compared to the baseline, were assessed. The mRS score at 3 months was evaluated in person in the clinic or by a structured telephone interview of the patients or caregivers by trained stroke physicians or trained study coordinators.

### Statistical analysis

The data are summarized as the mean (standard deviation) or median (interquartile range) values for continuous variables and as frequencies and percentages for categorical variables. Normality testing was performed with the Shapiro–Wilk test for continuous variables. Differences between the groups were assessed for significance using the Wilcoxon rank-sum test, Kruskal–Wallis test, analysis of variance, or Fisher’s exact test, as appropriate. Baseline characteristics and outcomes were compared between the groups. Logistic regression models were constructed for each binary outcome, and odds ratios (ORs) with 95% confidence intervals (CIs) for the mild anemia group and the moderate-to-severe anemia were calculated using the no anemia group as the reference. To determine a favorable shift in the mRS scores at 3 months, common ORs for a 1-point change in the mRS score were derived from ordinal logistic regression models. The following variables were included in the logistic regression models for covariate adjustment: age, sex, the baseline NIHSS score, the premorbid mRS score, the total number of device passes, Hb concentrations on admission, congestive heart failure, groin puncture to final angiography time, subarachnoid hemorrhage (SAH), and RBC transfusion. A logistic regression model was also constructed for moderate-to-severe anemia within 24 h after MT in the groups with two, three, and four or more device passes, using the group with one device pass as the reference. The following variables were included in the logistic regression model for covariate adjustment: age, sex, body weight, Hb concentrations on admission, creatine concentrations on admission, congestive heart failure, prestroke antiplatelet therapy, prestroke anticoagulant therapy, and first-line MT strategy. The receiver operating characteristic curve was used to test the sensitivity, specificity, and accuracy of our model. The area under the curve (AUC) was compared using the DeLong test. Restricted cubic spline analyses were used to detect potential linear or nonlinear dependence in the regression model and to allow for a flexible interpretation of the relation between the Hb concentrations as continuous data and favorable outcomes. Multivariable adjusted analyses with three knots (25th, 50th, and 75th percentiles) were used. The Jonckheere–Terpstra trend test was used to analyze the trend in Hb concentrations in relation to the total number of device passes. Missing data were handled using pairwise deletion. *p* < 0.05 was considered significant. Statistical analyses were performed using R version 4.0.3 (R Foundation for Statistical Computing, Vienna, Austria) and RStudio IDE version 1.3.959 (RStudio, Boston, MA, USA).

## Results

### Patients’ characteristics

The study flowchart is shown in Supplementary Figure S1. We analyzed 470 patients (217 women [46.2%], median age: 78 [interquartile range, 70–84] years; median NIHSS score: 18 [interquartile range, 12–24]). We found that 136 (28.9%) patients had mild anemia and 47 (10.0%) patients had moderate-to-severe anemia on admission. After MT, 168 (35.7%) patients had mild anemia and 136 (29%) patients had moderate-to-severe anemia.

Baseline characteristics of the no anemia, mild anemia, and moderate-to-severe anemia groups are shown in Table 1. Twenty-four of 287 (8.4%) patients without anemia on admission and 66 of 136 (48.5%) patients with mild anemia on admission showed postoperative moderate-to-severe anemia. The moderate-to-severe anemia group was older and had a higher rate of female sex than the no and mild anemia groups. Comorbid congestive heart failure and a history of stroke were more frequent in the moderate-to-severe anemia group than in the other groups. There were no significant differences in the frequency of chronic kidney disease or active cancer between the groups. No significant difference was found in the rate of intravenous thrombolysis between the groups.

**Table 1:** Baseline characteristics of the patients divided by Hb concentration within 24 h after mechanical thrombectomy.

	No anemia (*n* = 166)	Mild anemia (*n* = 168)	Moderate-to-severe anemia (*n* = 136)	*p* value*
Age, y	74 [65–80]	80 [72–84]	82 [73–86]	<0.001
Female sex	63 (38)	86 (51)	68 (50)	0.03
Body weight, kg	60 [52–70]	53 [46–62]	52 [45–60]	<0.001
Premorbid mRS score	0 [0–0]	0 [0–3]	0 [0–3]	<0.001
Baseline NIHSS score^†^	18 [11–24]	19 [13–24]	19 [12–24]	0.74
Systolic BP on admission, mmHg	153 [141–173]	151 [133–175]	150 [130–166]	0.08
ASPECTS^‡^	8 [6–9]	8 [6–10]	8 [7–10]	0.23
Medical history				
Atrial fibrillation^§^	111 (68)	108 (64)	85 (63)	0.57
Hypertension	116 (70)	126 (75)	101 (74)	0.54
Diabetes mellitus^∥^	36 (22)	41 (24)	27 (20)	0.64
Dyslipidemia	82 (49)	88 (52)	53 (39)	0.05
Congestive heart failure^¶^	28 (17)	43 (26)	50 (37)	0.001
Ischemic heart disease^**^	17 (10)	25 (15)	19 (14)	0.41
Chronic kidney disease	81 (49)	91 (54)	82 (60)	0.14
Previous stroke/TIA	25 (15)	42 (25)	34 (25)	0.04
Active cancer	4 (2)	8 (5)	11 (8)	0.08
Current smoker^††^	30 (18)	25 (15)	15 (11)	0.22
Prestroke antiplatelet therapy	31 (19)	46 (27)	29 (21)	0.16
Prestroke anticoagulant therapy	55 (33)	50 (30)	53 (39)	0.24
Anemic status before mechanical thrombectomy				
No anemia on admission	160 (96)	103 (61)	24 (18)	<0.001
Mild anemia on admission	6 (4)	64 (38)	66 (49)	
Moderate-to-anemia on admission	0 (0)	1 (1)	46 (34)	
Hb on admission, g/dL	14.6 [13.7–15.3]	12.7 [12.0–13.5]	11.1 [10.1–11.9]	<0.001
∆Hb, g/dL	−0.8 [−1.5−−0.3]	−1.3 [−2.0−−0.7]	−1.7 [−2.5−−1.0]	<0.001
RBC transfusion	0 (0)	2 (1)	30 (22)	<0.001
IVT	77 (46)	80 (48)	57 (42)	0.60
First-line MT strategy				
SR	50 (30)	61 (36)	45 (33)	0.03
CA	66 (40)	72 (43)	41 (30)	
Combined SR + CA	50 (30)	35 (21)	50 (37)	

Data are presented as the median [interquartile range] or *n* (%). ^*^Univariate comparisons between the no anemia, mild anemia, and moderate-to-severe anemia groups. ^**,†^One missing value. ^‡^Seven missing values. ASPECTS on non-contrast computed tomography (*n* = 166) and ASPECTS on diffusion-weighted magnetic resonance imaging (*n* = 297). ^§,∥^Three missing values. ^¶^Nine missing values. ^††^Five missing values.

mRS, modified Rankin Scale; NIHSS, National Institutes of Health Stroke Scale; BP, blood pressure; ASPECTS, Alberta Stroke Program Early Computed Tomographic Score; TIA, transient ischemic attack; RBC, red blood cell; IVT, intravenous thrombolysis; MT, mechanical thrombectomy; SR, stent retriever; CA, contact aspiration.

### Procedural outcomes and factors associated with anemia after MT

**Figure 1:**
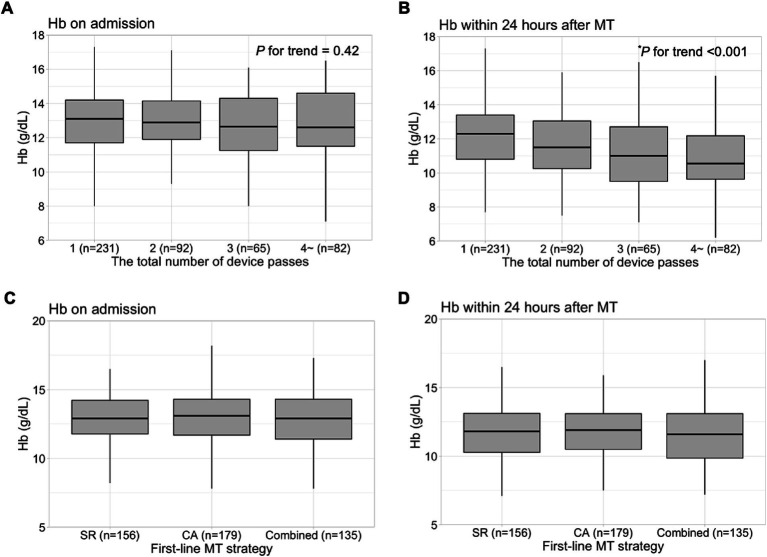
Hb concentrations on admission and within 24 h after MT. Hb concentrations on admission according to the total number of device passes **(A)** and Hb concentrations within 24 h after MT according to the total number of device passes **(B)**. Hb concentrations on admission according to the first-line MT strategy **(C)** and Hb concentrations within 24 h after MT according to the first-line MT strategy **(D)**. ^*^Jonckeheere–Terpstra trend test. Hb, hemoglobin; MT, mechanical thrombectomy; SR, stent retriever; CA, contact aspiration.

**Figure 2:**
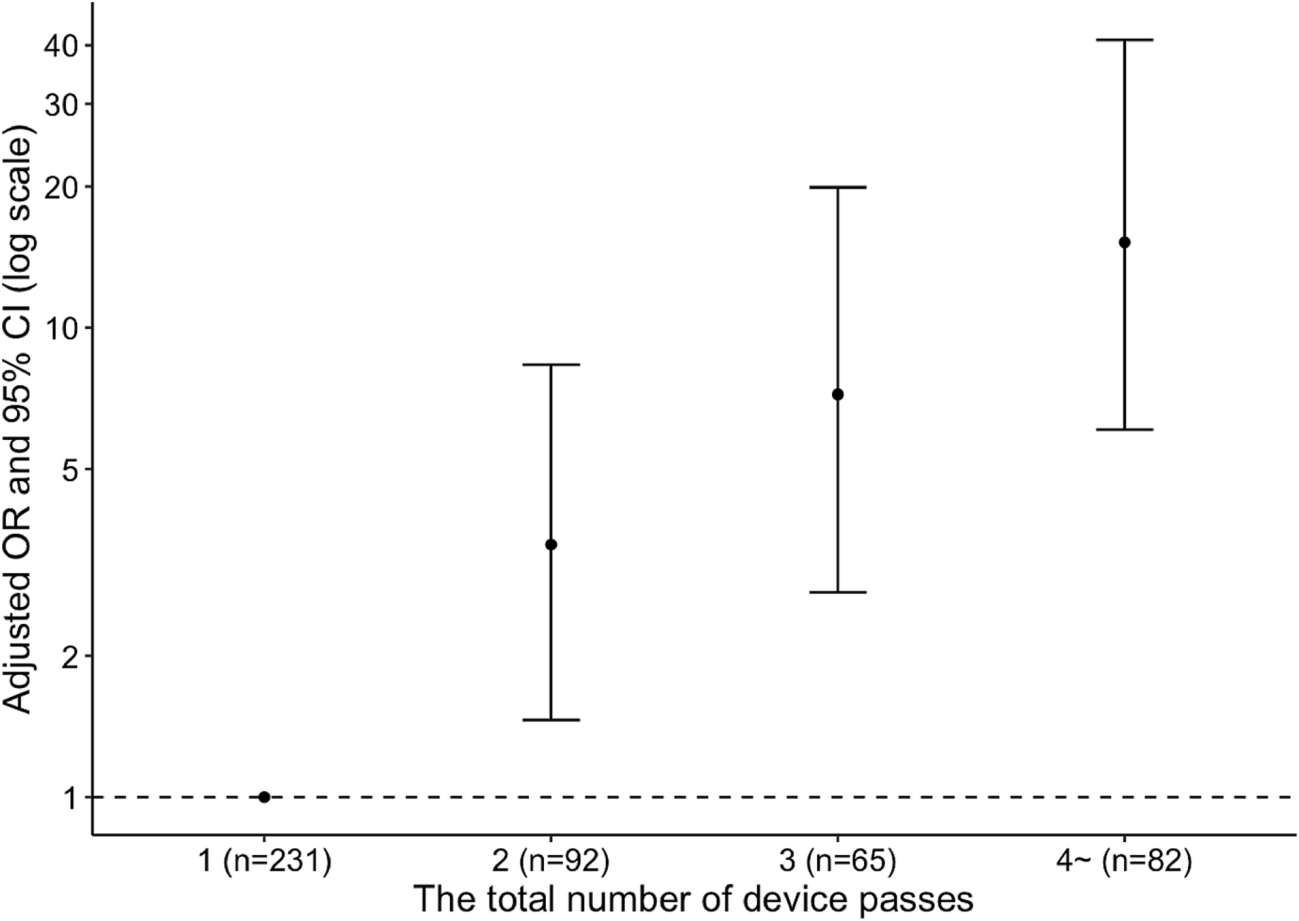
Adjusted odds ratios for moderate-to-severe anemia within 24 h after mechanical thrombectomy. OR, odds ratio; CI, confidence interval.

The moderate-to-severe anemia group had the longest time from groin puncture to first successful recanalization, the longest time from groin puncture to final angiography, the highest total number of device passes, the lowest rate of first pass effect, and the highest rate of perioperative subarachnoid hemorrhage among the three groups (Table 2). Hb concentrations after MT decreased with an increase in the number of device passes (*P* for trend <0.001) (Figure 1). In the multivariable analysis, moderate-to-severe anemia within 24 h after MT was more frequent in the group with two device passes (adjusted OR, 3.45; 95% CI, 1.46–8.34), three device passes (adjusted OR, 7.21; 95% CI, 2.73–19.90), and four or more device passes (adjusted OR, 15.20; 95% CI, 6.07–41.10) than the group with one device pass (Figure 2).

**Table 2:** Procedural outcomes.

	No anemia (*n* = 166)	Mild anemia (*n* = 168)	Moderate-to-severe anemia (*n* = 136)	*p* value*
Onset to groin puncture time, minutes	187 [126–410]	183 [110–422]	201 [116–430]	0.83
Groin puncture to first successful recanalization time, minutes	39 [27–63]	46 [30–78]	51 [36–89]	<0.001
Groin puncture to final angiography time, minutes	63 [38–89]	69 [43–117]	95 [60–141]	<0.001
Total number of device passes	1 [1–2]	2 [1–3]	2 [1–4]	<0.001
Final eTICI 2b–3	143 (86)	143 (85)	107 (79)	0.19
Final eTICI 2c–3	84 (51)	97 (58)	67 (49)	0.27
First pass effect	61 (37)	57 (34)	32 (24)	0.04
Symptomatic ICH	5 (3)	4 (2)	6 (4)	0.54
Any ICH	73 (44)	61 (36)	65 (48)	0.12
SAH	27 (16)	27 (16)	36 (27)	0.04

Data are presented as the median [interquartile range] or *n* (%). *Univariate comparisons between the no anemia, mild anemia, and moderate-to-severe anemia groups. eTICI, extended thrombolysis in cerebral infarction; ICH, intracranial hemorrhage; SAH, subarachnoid hemorrhage.

Regarding the first-line MT strategy, CA was the most frequently performed in the no anemia and mild anemia groups, while combined SR and CA was the most common in the moderate-to-severe anemia group (Table 1). However, there was no significant difference in Hb concentrations at admission or postoperative Hb concentrations between first-line MT strategies (Figure 1).

### Clinical outcomes

**Figure 3:**
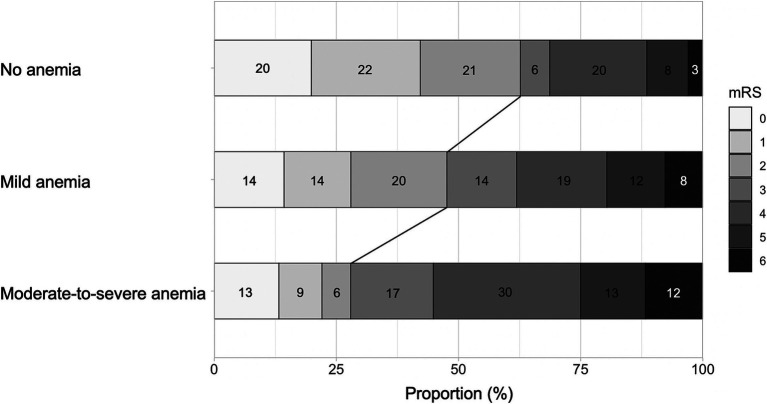
Distribution of the mRS score at 3 months. mRS, modified Rankin Scale.

The distribution of the mRS score at 3 months is shown in Figure 3. The moderate-to-severe anemia group was less likely to have a favorable outcome than the no anemia group (adjusted OR, 0.46; 95% CI, 0.26–0.81) (Table 3). In the sensitivity analysis of patients without mild to severe anemia on admission, the moderate-to-severe anemia group was also associated with a lower likelihood of a favorable outcome than the no anemia group, with an adjusted OR of 0.51 (95% CI, 0.27–0.96) (Supplementary Table S1).

**Table 3:** Clinical outcomes.

	No anemia (*n* = 166)	Mild anemia (*n* = 168)	Moderate-to-severe anemia (*n* = 136)	No anemia vs. mild anemia adjusted OR* (95% CI)	No anemia vs. moderate-to-severe anemia Adjusted OR^†^ (95% CI)
mRS score of 0–2 at 3 months^‡^	103 (69)	77 (62)	35 (36)	0.54 (0.26–1.12)	0.46 (0.26–0.81)
mRS score of 0–1 at 3 months^§^	69 (50)	45 (42)	26 (32)	0.76 (0.39–1.49)	0.84 (0.47–1.49)
Shift in mRS scores toward a better outcome	–	–	–	0.71 (0.43–1.16)	0.83 (0.56–1.22)
Mortality at 3 months	5 (3)	13 (8)	16 (12)	2.59 (0.66–10.21)	0.95 (0.39–2.33)
Neurological improvement at 7 days	103 (64)	98 (61)	69 (55)	0.82 (0.43–1.55)	0.88 (0.55–1.41)
Neurological deterioration at 7 days	6 (4)	8 (5)	9 (7)	1.64 (0.35–7.74)	1.56 (0.59–4.11)

Data are presented as *n* (%) or adjusted OR (95% CI). ^*^ORs with 95% confidence intervals for the mild anemia group were calculated using the no anemia group as the reference. ^†^ORs with 95% confidence intervals for the moderate-to-severe anemia group were calculated using the no anemia group as the reference. ^‡^Patients with a premorbid mRS score > 2 were excluded. ^§^Patients with a premorbid mRS score > 1 were excluded. Models for clinical outcomes were adjusted for age, sex, the baseline NIHSS score, the premorbid mRS score, congestive heart failure, Hb concentration on admission, the total number of device passes, groin puncture to final angiography time, SAH, and RBC transfusion. OR, odds ratio; CI, confidence interval; mRS, modified Rankin Scale; NIHSS, National Institutes of Health Stroke Scale.

**Figure 4:**
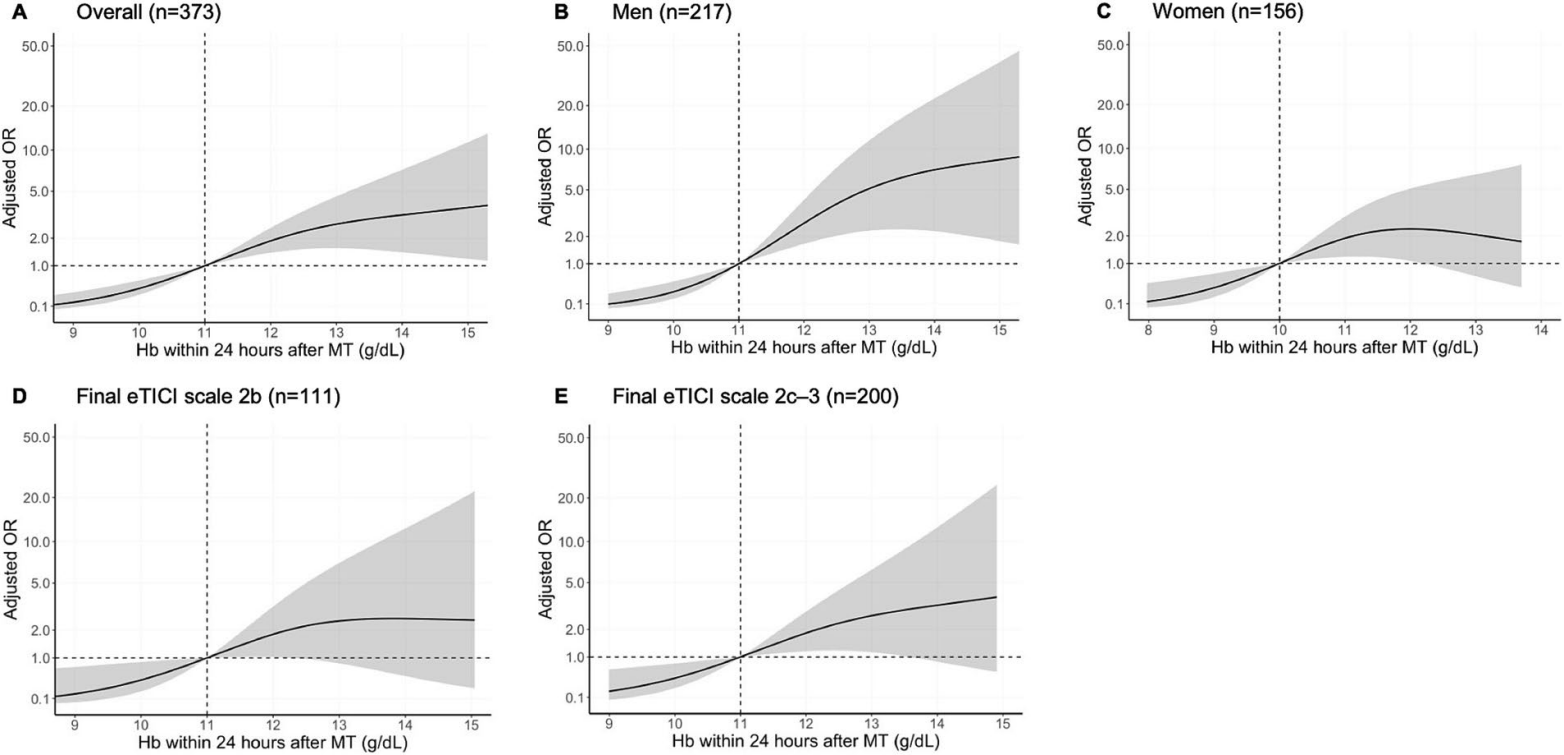
Association of a favorable outcome with Hb concentrations within 24 h after MT in a restricted cubic spline model. Multivariable adjusted odds ratios (adjusted OR; solid line) with 95% CIs (gray area) for the association of a favorable outcome with Hb concentrations within 24 h after MT in the overall patients **(A)**, in men **(B)**, in woman **(C)**, with a final eTICI grade of 2b **(D)**, and with a final eTICI grade of 2c–3 **(E)**, with Hb concentrations of 11, 11, 10, 11, and 11 g/dL as the reference values, respectively. eTICI, extended thrombolysis in cerebral infarction; Hb, hemoglobin; MT, mechanical thrombectomy.

The restricted cubic spline analysis showed that the adjusted OR for a favorable outcome was lower when Hb concentrations declined within 24 h after MT in the overall patients, as well in men, women, and patients who achieved final eTICI 2c–3 reperfusion (Figure 4).

### Predictive capability of Hb concentrations for the clinical outcome

The receiver operating characteristic curves for the evaluated variables are shown in Supplementary Figure S2. An analysis of the optimal performance cut-off point for a favorable outcome yielded a cut-off of 12.6 g/dL for Hb concentrations on admission, with a sensitivity of 67.5%, specificity of 55.6%, and AUC value of 0.629. With regard to Hb concentrations within 24 h after MT, cut-off scores were established at 11.3 g/dL, with a sensitivity of 71.6%, specificity of 57.2%, and AUC value of 0.684. With regard to the ΔHb, cut-off scores were established at −2.2 g/dL, with a sensitivity of 44.5%, specificity of 70.3%, and AUC value of 0.593. The AUC of Hb concentrations within 24 h after MT was significantly larger than that of Hb concentrations on admission (*p* = 0.001) and the ΔHb (*p* = 0.003).

## Discussion

The major finding in the present study was that patients with moderate-to-severe anemia within 24 h after MT had worse functional outcomes than those without anemia. Hb concentrations within 24 h after MT decreased as the number of device passes increased. Hb concentrations within 24 h after MT had a better predictive capability than the Hb concentrations on admission and the ∆Hb.

The prevalence of anemia on admission in the present study is consistent with that in previous studies (6, 7). The prevalence of mild to severe anemia within 24 h after MT is also similar to that in a previous study (7). There have been no previous reports on the frequency of moderate-to-severe anemia within 24 h after MT in the literature. The present study showed that postoperative moderate-to-severe anemia was relatively common in patients who underwent MT.

The baseline characteristics based on the Hb concentration on admission are comparable to those previously reported (6). Anemia after MT is reflected by the baseline Hb concentration in some patients. Therefore, the baseline characteristics and comorbidities that cause baseline anemia may have affected our results. In the present study, 8.4% of patients without anemia on admission and 48.5% of patients with mild anemia on admission showed postoperative moderate-to-severe anemia, which suggested that blood loss during the MT procedure exacerbated postoperative anemia.

Several pathophysiological mechanisms have been proposed to explain the impact of anemia on acute ischemic stroke. Oxygen supply to the brain is determined by cerebral blood flow, Hb concentration, and arterial oxygen saturation. In healthy patients, cerebral autoregulation maintains oxygen supply to the brain even when hemoglobin falls to about 8 g/dL (25), but it has been reported that cerebral autoregulation is impaired in stroke patients and remains impaired even when reperfusion is achieved by mechanical thrombectomy (26). Moreover, arterial blood pressure is likely to fluctuate in the acute stage of stroke (27), which suggests the importance of dynamic cerebral autoregulation during the early stage in the reperfusion phase in LVO (28). A recent study showed that an increased number of device passes was associated with infarct growth, even in patients with successful reperfusion (29). A previous study showed that decreased Hb concentrations during admission were associated with infarct growth (7). Postoperative anemia caused by multiple device passes might contribute to infarct growth and thus a poorer outcome. Hb concentrations may be of interest in neurocritical care after MT because a decline of Hb concentrations was associated with a lower odds of a favorable outcome even in patients who achieved a final eTICI grade of 2c–3.

This study showed that Hb concentrations within 24 h after MT had a higher predictive ability for favorable functional outcomes than Hb concentrations on admission or the ΔHb. Patients with moderate-to-severe anemia on admission have poor functional outcomes (6). Blood loss during the MT procedure might further worsen outcomes in patients with anemia on admission. In contrast, the predictive ability of the ΔHb for outcomes was moderate, which suggested that the effect of a decrease in Hb concentrations was inconsistent among patients. The present result suggests the importance of being aware of blood loss during the MT procedure, especially in patients with anemia on admission.

The present study has several limitations. First, the single-center study design with the small sample size in each group may have limited the statistical power. Second, the underlying cause of anemia was not identified, and other unknown confounders may have been present. Third, the catheters are connected to heparinized normal saline pressurized bags during MT, which may contribute to the hemodilution. The combined SR and CA technique uses a triple co-axial system, which may be prone to hemodilution because more heparinized normal saline pressurized bags are connected. However, the effect of hemodilution was not corrected in this study. Fourth, thrombectomy devices evolved during the period of enrollment in this study. Although these developments may increase the rate of first pass recanalization and reduce the risk of blood loss, they were not corrected. Fifth, this retrospective, observational study was not able to establish a causal relationship between moderate-to-severe anemia within 24 h after MT and the likelihood of a favorable outcome.

## Conclusion

Moderate-to-severe anemia within 24 h after MT is associated with a reduced likelihood of a favorable outcome after adjusting for baseline Hb concentrations.

## Data availability statement

The raw data supporting the conclusions of this article will be made available by the authors, without undue reservation.

## Ethics statement

The studies involving humans were approved by the Institutional Review Board of the National Cerebral and Cardiovascular Center. The studies were conducted in accordance with the local legislation and institutional requirements. The ethics committee/institutional review board waived the requirement of written informed consent for participation from the participants or the participants’ legal guardians/next of kin due to the retrospective, observational nature of the study.

## Author contributions

RI: Data curation, Formal analysis, Investigation, Writing – original draft. JK: Conceptualization, Data curation, Methodology, Writing – review & editing. KTa: Conceptualization, Data curation, Methodology, Supervision, Writing – review & editing. TY: Data curation, Supervision, Writing – review & editing. MS: Data curation, Writing – review & editing. SA: Data curation, Writing – review & editing. HIs: Data curation, Writing – review & editing. HIm: Supervision, Writing – review & editing. JN: Supervision, Writing – review & editing. HK: Supervision, Writing – review & editing. MI: Supervision, Writing – review & editing. KTo: Supervision, Writing – review & editing. MK: Supervision, Writing – review & editing.
