# Supplementary material for: Detrimental effect of anemia after mechanical thrombectomy on functional outcome in patients with ischemic stroke
Source: Front Neurol. 2023 Dec 19;14:1299891. doi: 10.3389/fneur.2023.1299891 (PMC10770243; doi:10.3389/fneur.2023.1299891)

**SUPPLEMENTAL MATERIAL**

**Detrimental effect of anemia due to post-mechanical thrombectomy on functional outcome in patients with stroke**

**Figure S1. Study flowchart.**

**
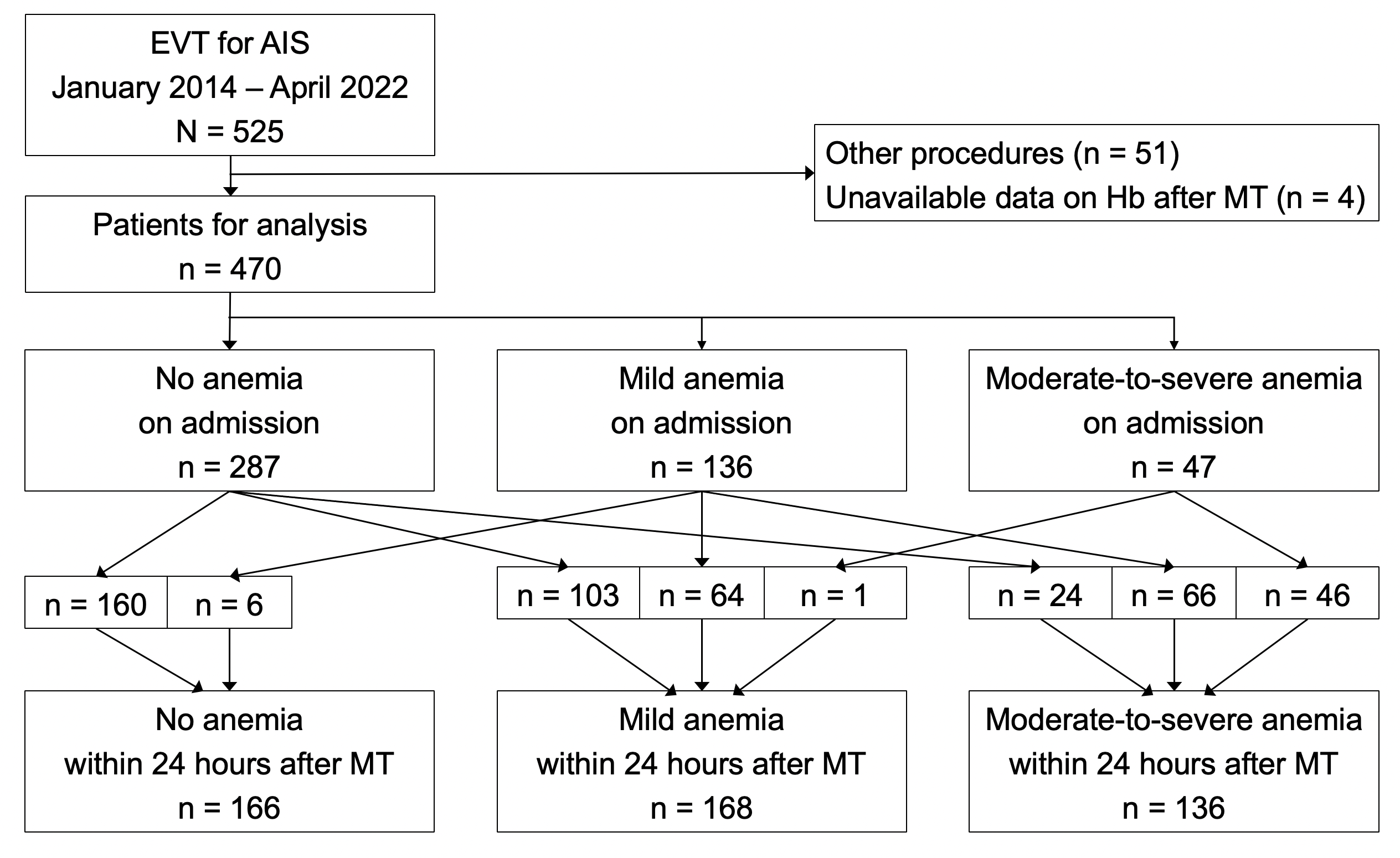
**

Abbreviations: EVT, endovascular therapy; AIS, acute ischemic stroke; MT, mechanical thrombectomy; Hb, hemoglobin.

**Table S1. Subgroup analysis: patients without mild to severe anemia on admission**

|  | **No anemia**  **(n=160)** | **Mild anemia**  **(n=103)** | **Moderate-to-severe anemia**  **(n=24)** | **No anemia vs. mild anemia**  **Adjusted OR***  **(95% CI)** | **No anemia vs. moderate-to-severe anemia**  **Adjusted OR**^†^  **(95% CI)** |
| --- | --- | --- | --- | --- | --- |
| mRS score of 0–2 at 3 months^‡^ | 99 (68) | 48 (60) | 5 (26) | 0.92 (0.49–1.71) | 0.51 (0.27–0.96) |
| mRS score of 0–1 at 3 months^§^ | 67 (50) | 27 (38) | 5 (31) | 0.68 (0.35–1.29) | 0.71 (0.37–1.36) |
| Shift in mRS scores toward a better outcome | – | – | – | 0.97 (0.61–1.54) | 0.78 (0.49–1.23) |
| Mortality at 3 months | 5 (3) | 7 (7) | 2 (8) | 1.56 (0.43–5.58) | 1.18 (0.40–3.52) |
| Neurological improvement at 7 days | 99 (64) | 61 (64) | 12 (50) | 1.11 (0.62–1.97) | 0.93 (0.56–1.54) |

Data are presented as n (%) or adjusted OR (95% CI).

*ORs with 95% confidence intervals for the mild anemia group were calculated using the no anemia group as the reference.

^†^ORs with 95% confidence intervals for the moderate to severe anemia group were calculated using the no anemia group as the reference.

^‡^Patients with a premorbid mRS score >2 were excluded.

^§^Patients with a premorbid mRS score >1 were excluded.

Models for clinical outcomes were adjusted for age, sex, the baseline NIHSS score, the premorbid mRS score, the number of total device passes, and congestive heart failure.

Abbreviations: OR, odds ratio; CI, confidence interval; mRS, modified Rankin Scale; NIHSS, National Institutes of Health Stroke Scale.

**Figure S2. ROC curves.**

The red line represents the ROC curve of Hb on admission for a favorable outcome. The green line represents the ROC curve of Hb within 24 hours after MT for a favorable outcome. The blue line represents the ROC curve of the ∆Hb for a favorable outcome.

Abbreviations: ROC, receiver operating characteristic; Hb, hemoglobin; MT, mechanical thrombectomy.


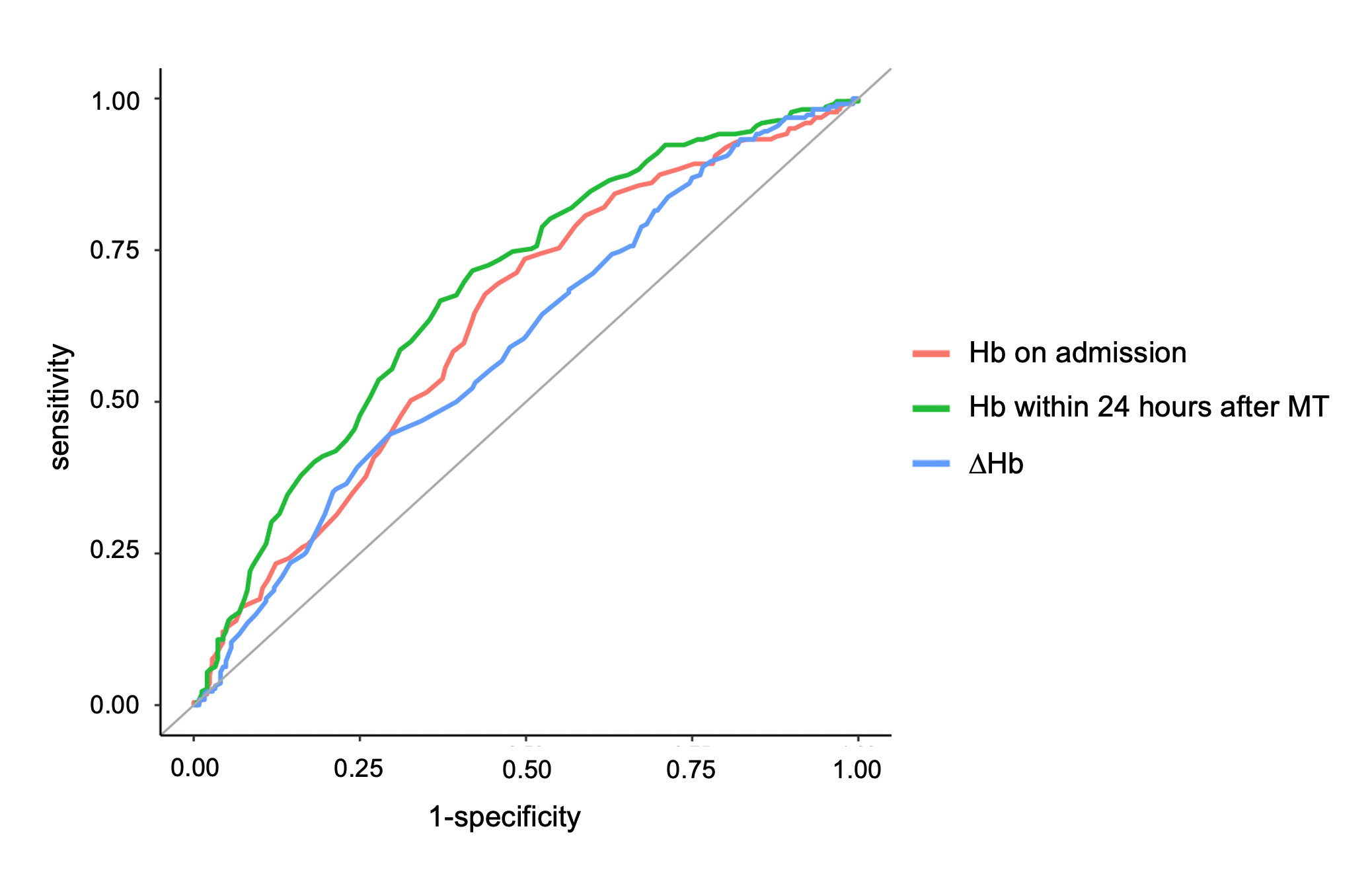

Supplement: Supplementary file 1 [file Data_Sheet_1.docx]
